# Supplementary material for: Genome-wide association meta-analysis identifies GP2 gene risk variants for pancreatic cancer
Source: Nat Commun. 2020 Jun 24;11:3175. doi: 10.1038/s41467-020-16711-w (PMC7314803; doi:10.1038/s41467-020-16711-w)
Supplement: Supplementary file 1 — Supplementary Information [file 41467_2020_16711_MOESM1_ESM.pdf]

SUPPLEMENTARY INFORMATION

Genome-wide association meta-analysis identifies *GP2* gene risk variants for pancreatic cancer.

Lin et al.

Supplementary Tables

Supplementary Table 1. Characteristics of the study participants. p.3-4

Supplementary Table 2. Four loci with suggestive significance in the meta-analysis of three Japanese GWASs. p. 5

Supplementary Table 3. Associations between selected SNPs at 16p12.3 and pancreatic cancer risk in the replication study. p. 6-7

Supplementary Table 4. Association results for rs4383153, located at 16p12.3, in the PanScan GWAS. p. 8

Supplementary Table 5. Associations between the top 3 SNPs for pancreatic cancer and T2D risk and quantitative traits in the Japanese GWAS. p. 9

Supplementary Table 6. Genes with $P<1\times10^{-4}$ based on the gene-based tests using MAGMA. p. 10

Supplementary Table 7. Primers used in the generation of GP2_V282M PaTu 8988s cell line and quantitative Real-time PCR. p. 11

Supplementary Figures

Supplementary Figure 1 Q-Q plot for the P values in the meta-analysis. p. 12

Supplementary Figure 2 Regional association plots for the six loci identified in the meta-analysis. p.13-18

Supplementary Figure 3 LD maps of 10 SNPs with genome-wide significance at 16p12.3 based on 1000 Genomes (a) JPT and (b) CEU subjects. p. 19

Supplementary Figure 4 MR analysis with the MR-Egger method for evaluating the relationship between T2D and pancreatic cancer in the Japanese population. p. 20-21

Supplementary Figure 5 MR analysis of the relationship between blood glucose, BMI, and pancreatic cancer in the Japanese population. p. 22-25

Supplementary Figure 6 Manhattan plot for the gene-based analysis. p.26

Supplementary Figure 7 Q-Q plot for the *P* values in the gene-based analysis. p.27

Supplementary Figure 8 GP2 differential gene expression illustrated across tissues. p. 28

Supplementary Figure 9 Relative mRNA expression levels of genes in *p53* pathway. p. 29

Supplementary Figure 10 Relative mRNA expression levels of genes in *KRAS* signaling pathway. p. 30

Supplementary Notes

Supplementary Note 1. Discussions on results of transient transfect experiments. p. 31-32

Supplementary Note 2. Additional details on the Biobank Japan Project and the population-based cohort studies. p. 33-35

Supplementary Note 3. Additional details on studies included in the replication analysis. p. 36-38

Supplementary References p.39

Supplementary Table 1. Characteristics of the study participants

Phase	Study name	Group	Source	Sample size[a]	Age (Mean±SD)	Male (%)
GWAS	JaPAN	Case	Hospital	943	64.7 ± 10.1	62.6
		Control	Hospital, Screening facility	3,057	52.1 ± 11.8	49.3
	NCC	Case	Hospital	674	62.7 ± 9.2	60.2
		Control	Volunteers, Health checkup program	674	43.6 ± 10.0	63.6
	BBJ[b]	Case	Hospital	422	66.3 ± 10.0	66.4
		Control	Population-based cohort participants	28,861	56.3 ± 10.0	39.4
Replication study	JaPAN	Case	Hospital	507	66.3 ± 9.1	53.3
		Control	Hospital, Screening facility	879	61.8 ± 11.2	51.2
	JEPA vs HERPACC	Case	Hospital	299	70.8 ± 8.7	57.2
		Control	Hospital, Screening facility	934	47.8 ± 16.3	52.2
	JMICC	Case	Population-based cohort participants	82	61.3 ± 7.0	59.8
		Control	Population-based cohort participants	249	61.2 ± 6.9	59
	JPHC	Case	Population-based cohort participants	85	57.5±7.9	44.7
		Control	Population-based cohort participants	2,493	53.9 ± 7.9	35.4

Yale- Shanghai	Case	37 metropolitan hospitals diagnosing pancreatic cancer	770	59.6 ± 9.9	56.5
	Control	Shanghai Residents Registry population random sample	744	59.9 ± 9.9	56.0
MEC	Case	Population-based cohort participants	183	78.0 ± 8.1	42.1
	Control	Population-based cohort participants	3,597	68.9 ± 8.4	53.0

[a] Sample size indicates the number of samples that passed quality control and were subjected to genome-wide meta-analysis.

[b] In the BBJ GWAS, control subjects were recruited from population-based cohort studies, including J-MICC, JPHC, ToMMo, and IMM.

Supplementary Table 2. Four loci with suggestive significance in the meta-analysis of three Japanese GWASs

SNP	Locus	Chr	Position	Gene	Alleles		Study	r^2	RAF		OR (95%CI)	*P* value	I^2	HetP value
					Risk	Non-risk			Case	Control				
rs3737136	1p13.2	1	113060432	*WNT2B*	G	A	JaPAN	0.767	0.467	0.445	1.14 (1.01-1.28)	0.036		
							NCC	0.875	0.498	0.466	1.16 (0.98-1.37)	0.079		
							BBJ	0.971	0.572	0.477	1.46 (1.27-1.68)	1.15E-07		
							Meta-analysis				1.24 (1.14-1.34)	1.35E-07	74.3	0.020
rs35067842	2p12	2	79568318	*REG3A*	C	A	JaPAN	0.869	0.633	0.599	1.23 (1.09-1.39)	6.09E-04		
				LOC101927987			NCC	0.890	0.622	0.570	1.28 (1.08-1.50)	0.003		
							BBJ	0.884	0.643	0.596	1.21 (1.04-1.40)	0.015		
							Meta-analysis				1.24 (1.14-1.34)	3.28E-07	0	0.883
rs6809193	3p12.3	3	76479914	*ZNF717*	G	A	JaPAN	0.966	0.694	0.663	1.20 (1.06-1.34)	0.003		
				ROBO2			NCC	0.970	0.665	0.632	1.15 (0.98-1.35)	0.089		
							BBJ	0.964	0.723	0.650	1.38 (1.18-1.61)	4.45E-05		
							Meta-analysis				1.23 (1.14-1.33)	4.33E-07	34.2	0.219
rs7855466	9q34.2	9	136121303	*OBP2B*	T	C	JaPAN	0.972	0.461	0.395	1.32 (1.18-1.47)	4.50E-07		
				ABO			NCC	0.977	0.435	0.397	1.19 (1.01-1.40)	0.037		
							BBJ	0.980	0.421	0.395	1.09 (0.95-1.25)	0.223		
							Meta-analysis				1.22 (1.13-1.31)	2.38E-07	56.7	0.100

OR values represent the increased risk of pancreatic cancer per risk allele copy for each SNP. Th r^2 value shows the imputation quality score. OR and *P* values were calculated by logistic regression analysis and fixed effects inverse variance-weighted meta-analysis (two-sided). Chr, chromosome. RAF, risk allele frequency. HetP value, *P* value from test of heterogeneity

Supplementary Table 3. Associations between selected SNPs at 16p12.3 and pancreatic cancer risk in the replication study

SNP	Chr	Position	Alleles		Study	r^2	RAF		OR (95% CI)	*P* value	I^2	HetP value
			Risk	Non-risk			Case	Control				
rs78193826	16	20328666	T	C	JaPAN	-	0.091	0.075	1.26 (0.94-1.68)	1.20E-01		
					JEPA vs HERPACC	-	0.104	0.080	1.35 (0.98-1.86)	6.90E-02		
					J-MICC	-	0.122	0.062	2.11 (1.15-3.84)	1.52E-02		
					JPHC	0.985	0.062	0.070	0.87 (0.46-1.65)	6.62E-01		
					Yale Shanghai	0.981	0.105	0.076	1.43 (1.11-1.84)	5.53E-03		
					MEC	0.998	0.063	0.074	0.85 (0.56, 1.31)	4.70E-01		
					Meta-analysis				1.29 (1.11-1.49)	6.28E-04	40.0	0.139
rs117267808	16	20323168	A	G	JaPAN	-	0.091	0.075	1.25 (0.93-1.66)	1.34E-01		
					JEPA vs HERPACC	-	0.104	0.080	1.36 (0.98-1.87)	6.25E-02		
					J-MICC	-	0.122	0.058	2.33 (1.25-4.34)	7.59E-03		
					JPHC	0.983	0.059	0.070	0.81 (0.42-1.57)	5.27E-01		
					Yale Shanghai	1.000	0.106	0.077	1.42 (1.11-1.83)	5.84E-03		
					MEC	0.999	0.063	0.074	0.85 (0.56, 1.31)	4.70E-01		
					Meta-analysis				1.29 (1.11-1.49)	6.42E-04	48.4	0.084
rs73541251	16	20331737	G	C	JaPAN	-	0.091	0.071	1.34 (1.00-1.79)	5.01E-02		
					JEPA vs HERPACC	-	0.094	0.077	1.26 (0.90-1.76)	1.70E-01		
					J-MICC	-	0.122	0.060	2.18 (1.19-3.98)	1.17E-02		
					JPHC	0.995	0.066	0.072	0.90 (0.48-1.69)	7.47E-01		
					Yale Shanghai	0.989	0.106	0.077	1.43 (1.11-1.84)	5.00E-03		
					MEC	0.960	0.065	0.074	0.87 (0.57, 1.34)	5.38E-01		
					Meta-analysis				1.30 (1.12-1.50)	4.28E-04	37.7	0.155

rs4632135	16	20337884	C	T	JaPAN	-	0.092	0.071	1.34 (1.00-1.79)	4.63E-02		
					JEPA vs HERPACC	-	0.090	0.078	1.19 (0.85-1.66)	3.12E-01		
					J-MICC	-	0.122	0.062	2.11 (1.15-3.84)	1.52E-02		
					JPHC	1.000	0.071	0.072	0.98 (0.53-1.78)	9.39E-01		
					Yale Shanghai	0.999	0.106	0.078	1.42 (1.10-1.82)	6.17E-03		
					MEC	0.936	0.067	0.074	0.91 (0.59, 1.41)	6.79E-01		
					Meta-analysis				1.29 (1.12-1.49)	5.52E-04	24.6	0.250

OR values represent the increased risk of pancreatic cancer per risk allele copy for each SNP. OR and *P* values were calculated by logistic regression analysis and fixed effects inverse variance-weighted meta-analysis (two-sided). Chr, chromosome. RAF, risk allele frequency. HetP value. *P* value from test of heterogeneity

r^2 and info scores show the imputation quality scores.

Supplementary Table 4. Association results for rs4383153, located at 16p12.3, in the PanScan GWAS

SNP	Chr	Position	Alleles		Study (dbGap accession)	Reference	N		*OR* (95% CI)	*P* value
			Risk	Non-risk			Case	Control		
rs4383153	16	20338622	A	G	PanScan 1 (pha002874.1)	Amundadottir et al. (Nat Genet 41:986 (2009))	1896	1939	0.99 (0.71-1.39)	0.962
					PanScan 1 and PanScan 2 (pha002889.1)	Li et al. (Carcinogenesis 33:1384 (2012))	3851	3934	1.04 (0.83-1.32)	0.725

OR values represent the increased risk of pancreatic cancer per risk allele copy for each SNP. OR and *P* values were calculated by logistic regression analysis (two-sided). Chr, chromosome.

Supplementary Table 5. Associations between the top 3 SNPs for pancreatic cancer and T2D risk and quantitative traits in the Japanese GWAS

SNP	Chr	Position	Alleles		T2D (Suzuki et al., N=191764)				HbA1c (Kanai et al., N=42790)			Blood glucose (Kanai et al., N=93146)		
			Risk	Non-risk	RAF	OR (95%CI)	*P*	HetP value	RAF	Beta (SE)	*P*	RAF	Beta (SE)	*P*
rs78193826	16	20328666	T	C	0.078	1.12 (1.08-1.15)	4.57E-12	0.0073	0.077	0.051 (0.013)	8.34E-05	0.077	0.028 (0.009)	0.0012
rs117267808	16	20323168	A	G	0.078	1.12 (1.08-1.16)	2.99E-12	0.0053	0.077	0.051 (0.013)	9.07E-05	0.077	0.028 (0.009)	0.0013
rs73541251	16	20331737	G	C	0.077	1.12 (1.08-1.15)	1.23E-11	0.0059	0.076	0.051 (0.013)	7.71E-05	0.076	0.029 (0.009)	0.0010

OR values represent the increased risk of T2D per risk allele copy for each SNP. *P* values were calculated by fixed effects inverse variance-weighted meta-analysis and linear regression analysis (two-sided). Chr, chromosome. RAF, risk allele frequency. HetP value, *P* value from test of heterogeneity

Beta values represent the change in the rank-based inverse normal transformed values of HbA1c and the blood glucose levels per risk allele copy for each SNP.

Supplementary Table 6. Genes with P<1×10^{-4} based on the gene-based tests using MAGMA

EntrezID	GeneSymbol	Chromosome	Start	End	*P*
7482	*WNT2B*	1	113010040	113063910	1.92E-07
3856	*KRT8*	12	53290971	53343650	1.33E-06
2813	*GP2*	16	20320896	20339684	2.00E-06
338	*APOB*	2	21224301	21266945	3.92E-06
54879	*ST7L*	1	113066140	113162040	6.52E-06
333926	*PPM1J*	1	113252616	113257950	1.59E-05
151194	*METTL21A*	2	208473839	208490652	2.30E-05
29989	*OBP2B*	9	136080666	136084714	2.77E-05
316	*AOX1*	2	201450731	201536218	3.58E-05
4343	*MOV10*	1	113217048	113243368	5.73E-05
829	*CAPZA1*	1	113162075	113214241	6.04E-05
646625	*URAD*	13	28552243	28562774	7.11E-05
1385	*CREB1*	2	208394616	208470284	7.83E-05
91351	*DDX60L*	4	169277886	169401665	8.49E-05
9353	*SLIT2*	4	20254566	20622184	9.57E-05

Genome-wide significance: P<2.84×10^{-6} after the Bonferroni correction. *P* values were calculated by the MAGMA software (two sided).

Supplementary Table 7. Primers used in the generation of GP2_V282M PaTu 8988s cell line and quantitative real-time PCR

	Application	Forward (5'-3')	Reverse (5'-3')
KLK7	qRT-PCR	CCCCTGGAACCACCTGTACT	CATGAGGTCAGAGGGAAAGG
KLK8	qRT-PCR	GGGTCCGAATCAGTAGGTGA	GAGCAGGAACATCCACGTCT
BMPR1B	qRT-PCR	GTCCAAAGGTCTTGCGTTGT	ACAGGCAACCCAGAGTCATC
TP53	qRT-PCR	GCGAGCACTGCCCAACAACA	GGATCTGAAGGGTGAAATATTCT
DDB2	qRT-PCR	CAAGGTTAGGGTTGGAGCAGG	AACTCTGGAGCAGTCTCTGGAGG
CDKN1A	qRT-PCR	TACCCTTGTGCCTCGCTCAG	GGCGGATTAGGGCTTCCTCT
sgGP2	CRISPR	CACCGATCACGTTGATTTGAGCAGCTGG	AAACCCAGCTGCTCAAATCAACGTGATC
GP2 repair template	CRISPR	AGAAGTTACACCTTTGCCCCTGTCACCCTCGCAGCTGCTCAAATC AACGTGATTCCACCATCCACATGGAGGAGAATGGGCAGTCCTCG GAAAGCCGGT	NA
GP2 genotyping	CRISPR	CTTTGCCCCTGTCACCCTCC	CTGAGAACCGGCTTTCCGAG

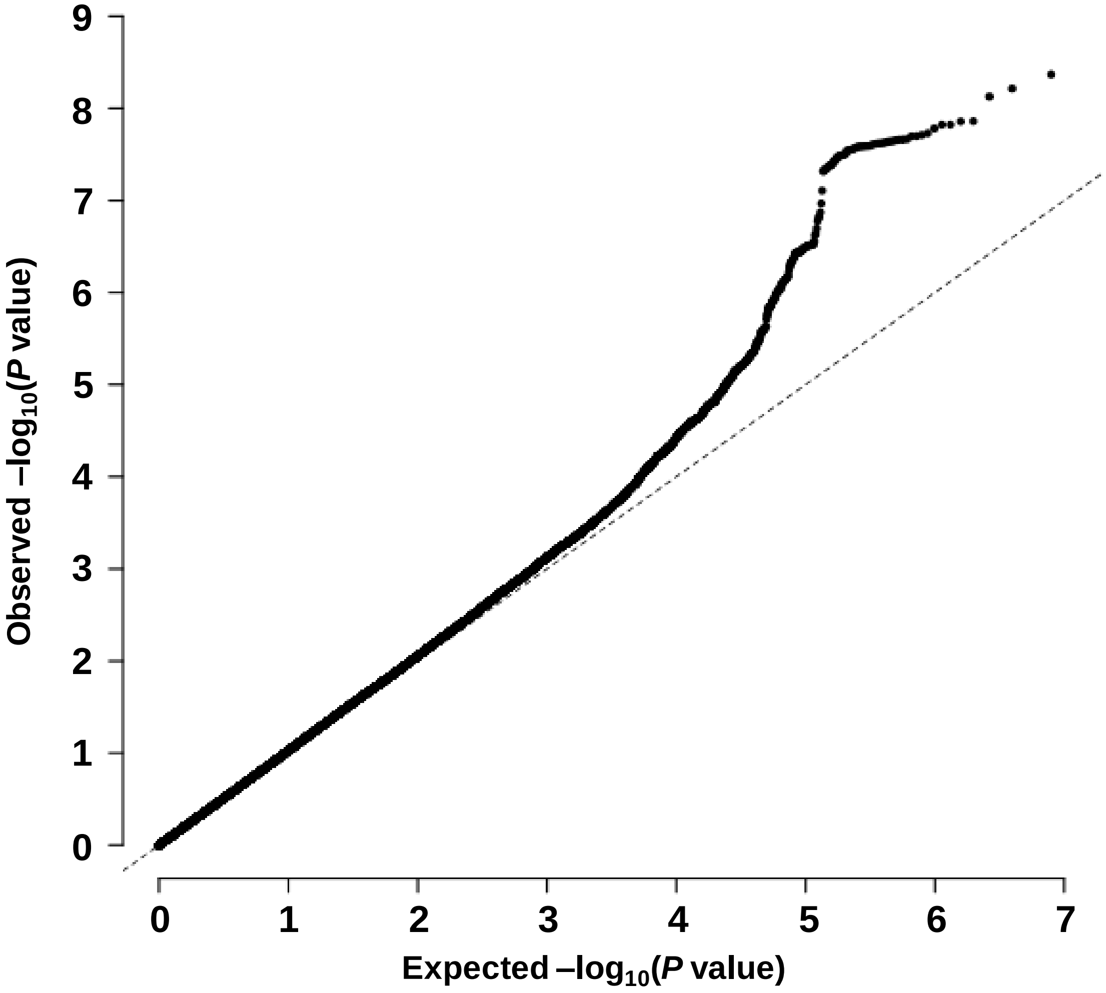

Supplementary Figure 1. Q-Q plot for the P values in the meta-analysis. The vertical and horizontal axes indicate the observed and expected $-\log_{10}$(P value) for tests of association between SNPs and pancreatic cancer, respectively.

a.

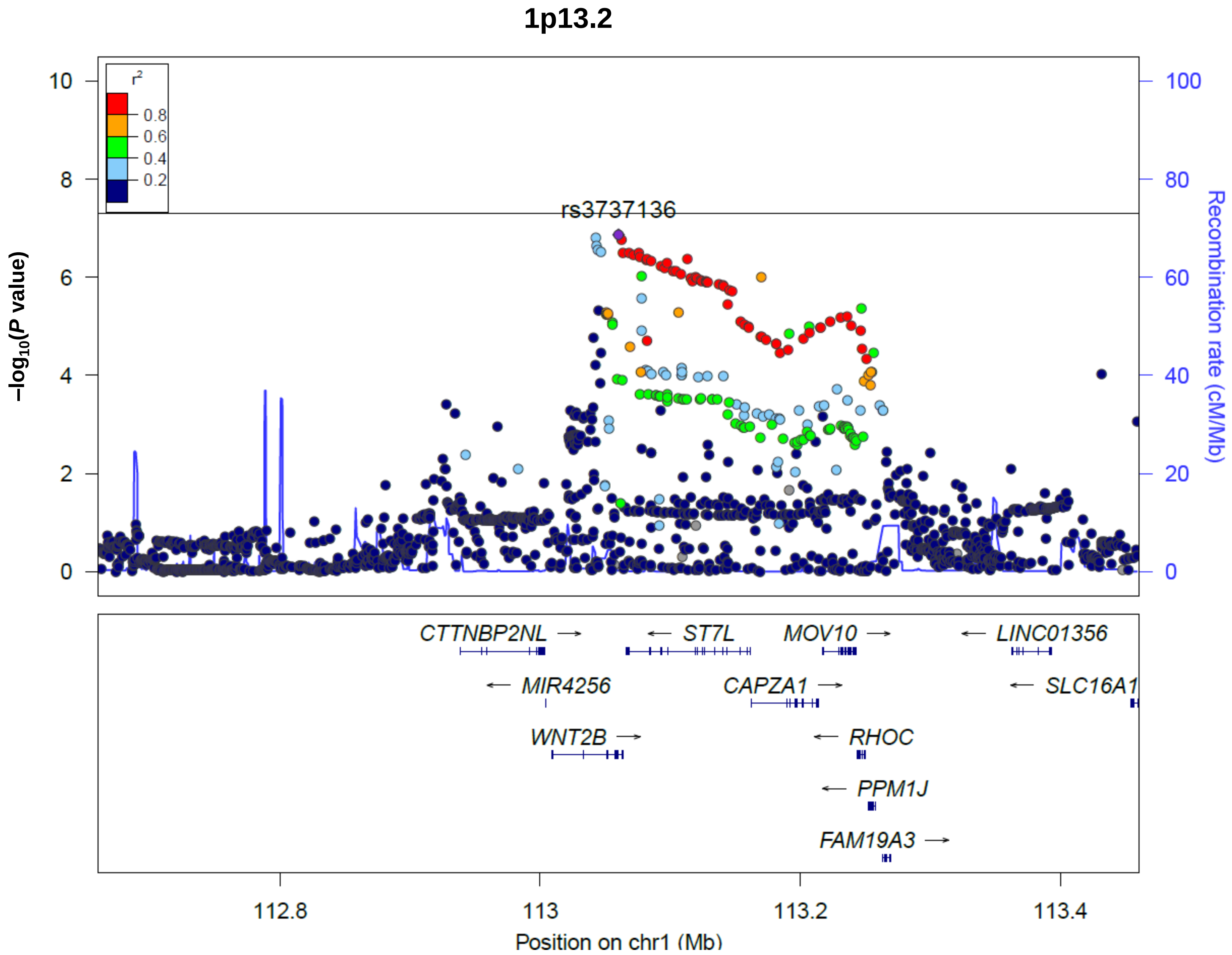

1p13.2
r²
0.8
0.6
0.4
0.2
rs3737136
10
8
6
4
2
0
−log10(P value)
100
80
60
40
20
0
Recombination rate (cM/Mb)
CTTNBP2NL →
← ST7L
MOV10 →
← LINC01356
← MIR4256
CAPZA1 →
← SLC16A1
WNT2B →
← RHOC
← PPM1J
FAM19A3 →
112.8
113
113.2
113.4
Position on chr1 (Mb)

b.

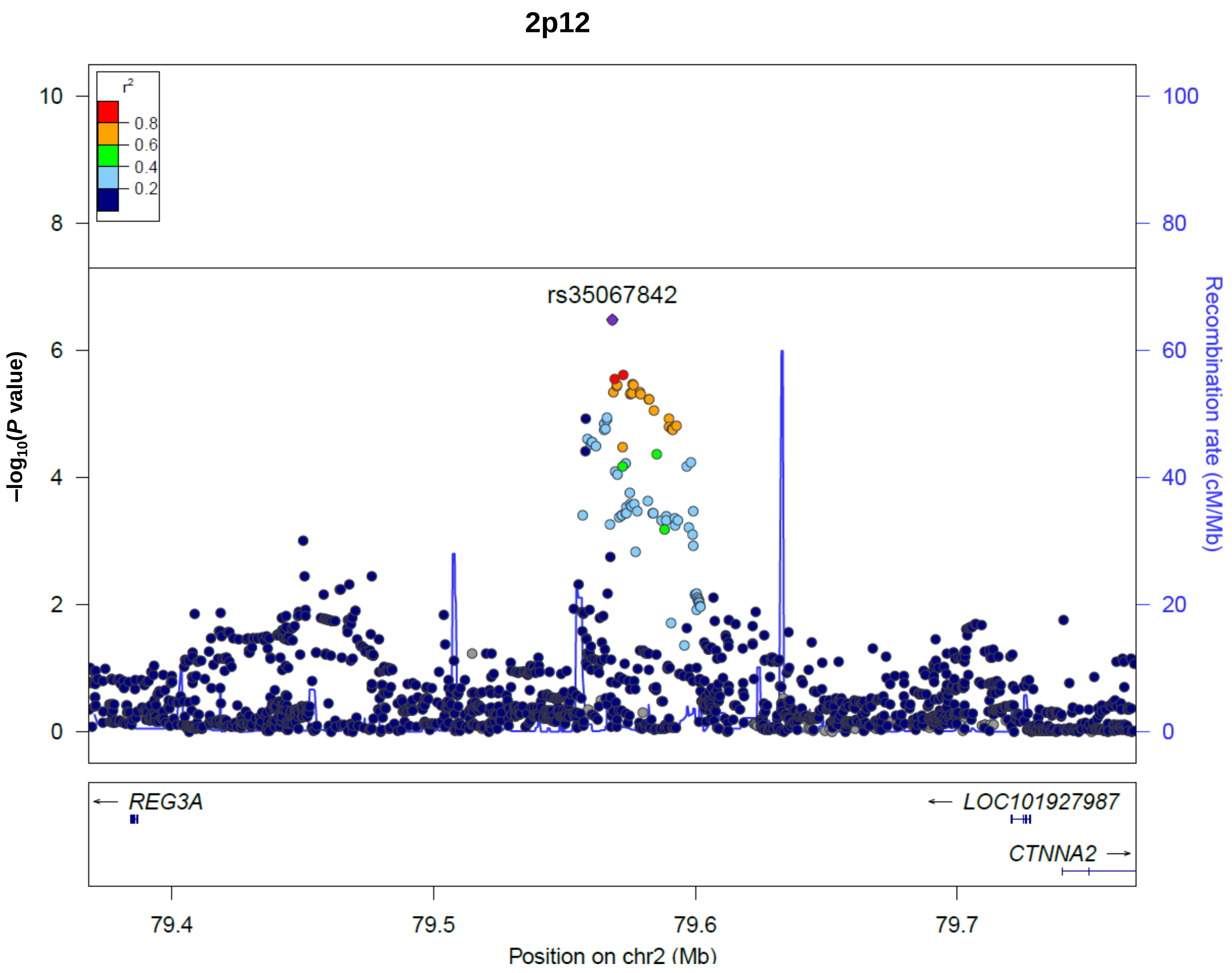
2p12
r²
0.8
0.6
0.4
0.2
10
8
6
4
2
0
−log10(P value)
100
80
60
40
20
0
Recombination rate (cM/Mb)
rs35067842
REG3A
LOC101927987
CTNNA2
79.4
79.5
79.6
79.7
Position on chr2 (Mb)

c..

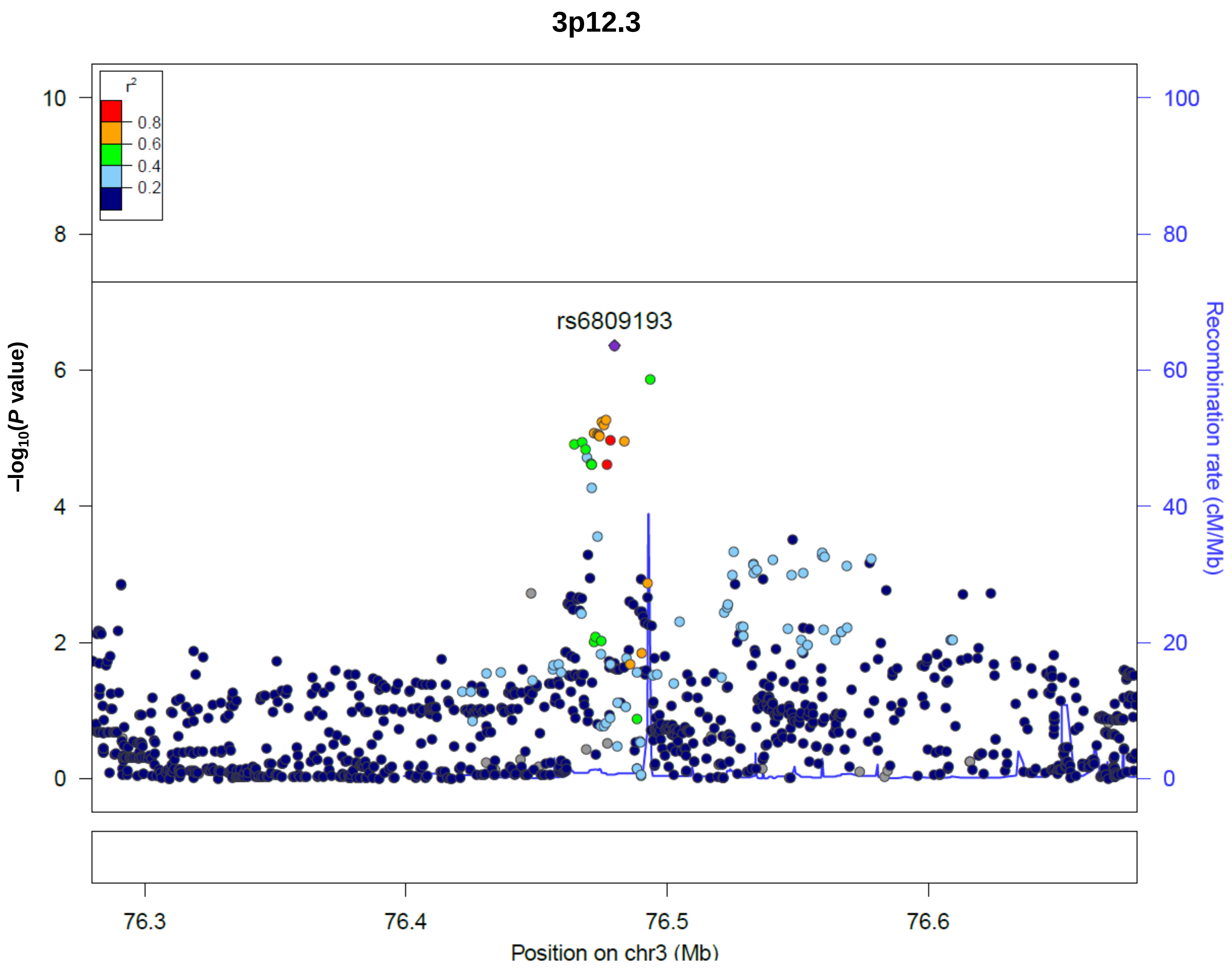
3p12.3
r²
0.8
0.6
0.4
0.2
rs6809193
10
8
6
4
2
0
−log10(P value)
100
80
60
40
20
0
Recombination rate (cM/Mb)
76.3
76.4
76.5
76.6
Position on chr3 (Mb)

d.

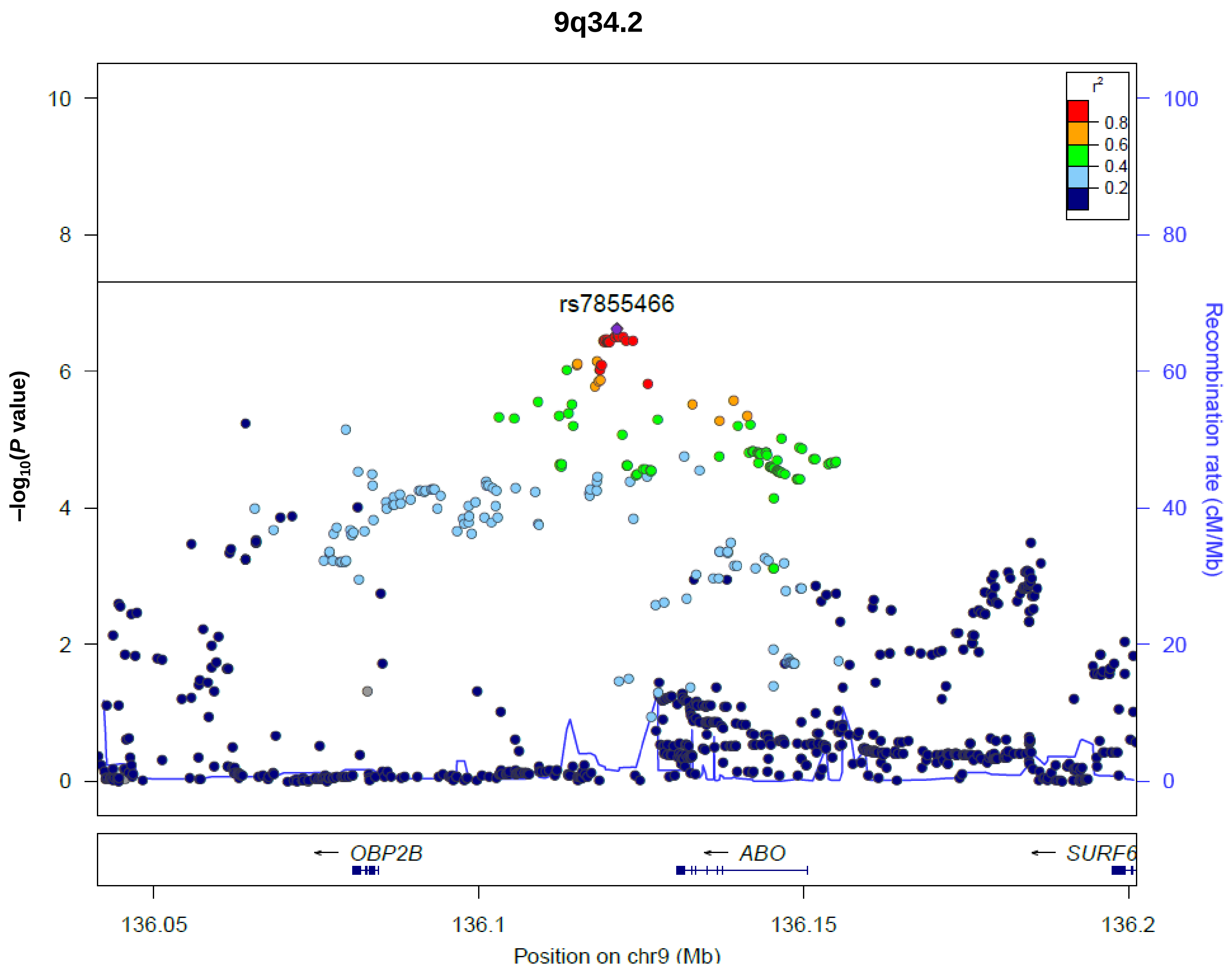

9q34.2
rs7855466
r²
0.8
0.6
0.4
0.2
−log10(P value)
Recombination rate (cM/Mb)
10
8
6
4
2
0
100
80
60
40
20
0
OBP2B
ABO
SURF6
136.05
136.1
136.15
136.2
Position on chr9 (Mb)

e.

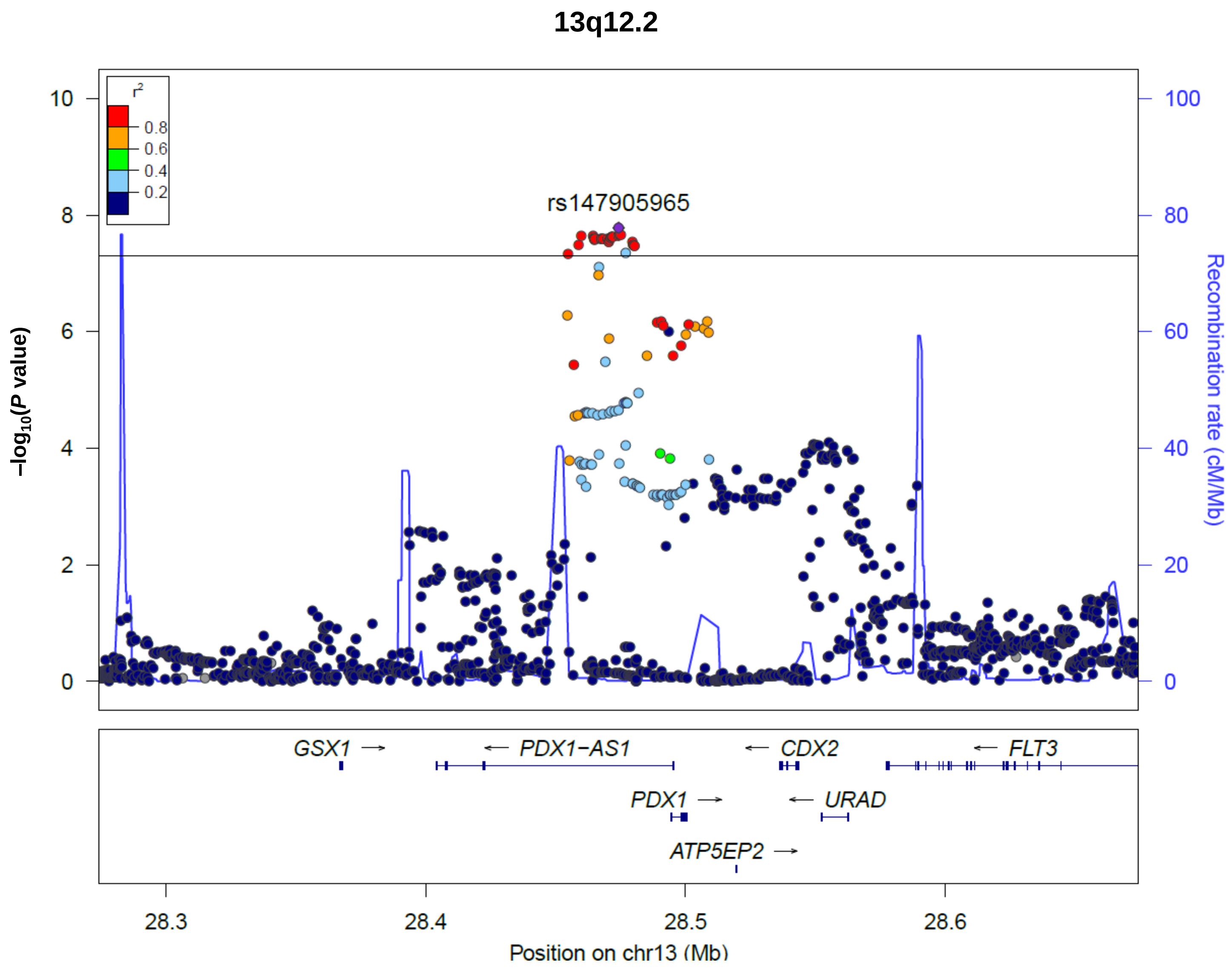
13q12.2
r²
0.8
0.6
0.4
0.2
rs147905965
10
8
6
4
2
0
−log10(P value)
100
80
60
40
20
0
Recombination rate (cM/Mb)
GSX1 →
← PDX1−AS1
← CDX2
← FLT3
PDX1 →
← URAD
ATP5EP2 →
28.3
28.4
28.5
28.6
Position on chr13 (Mb)

f.

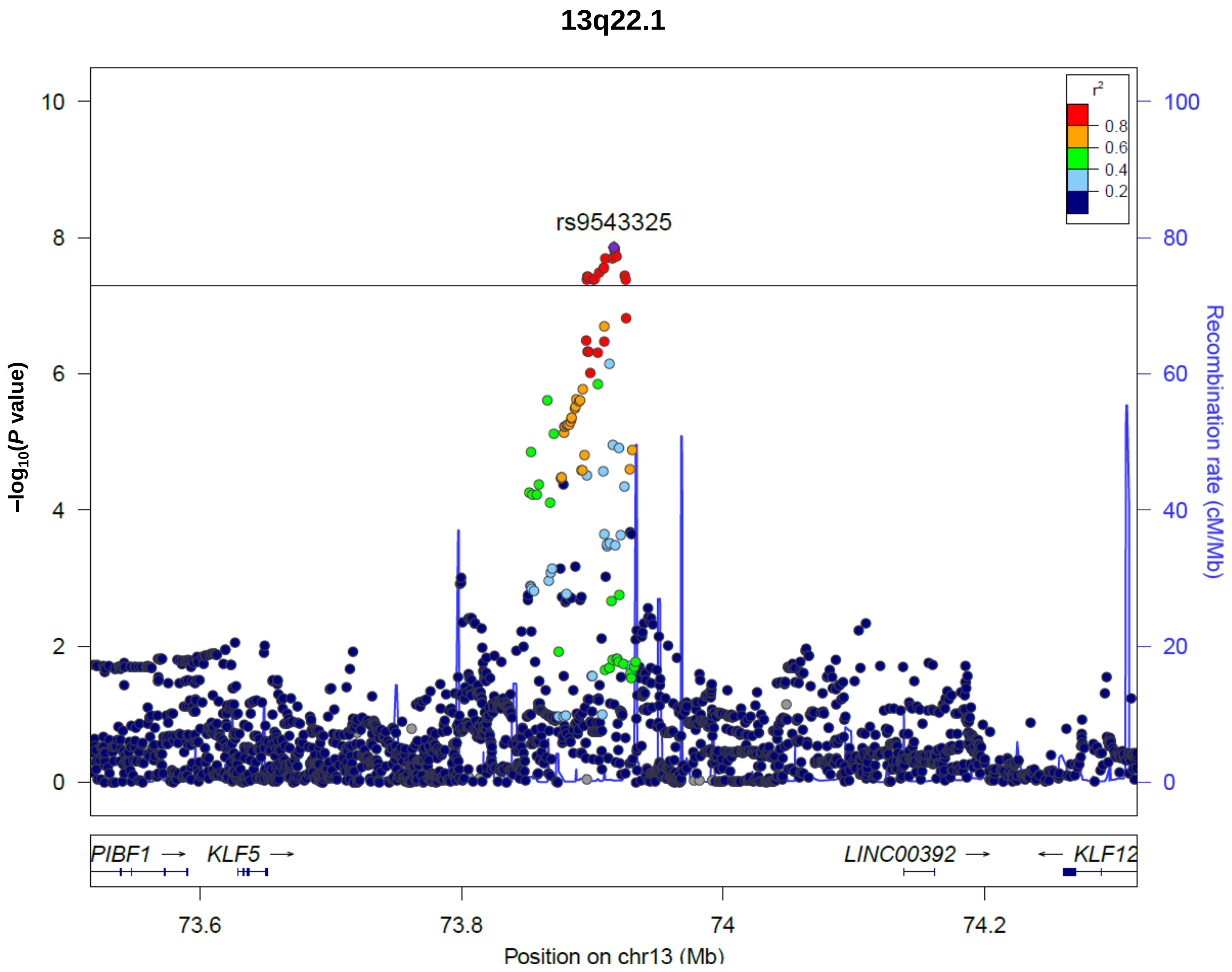

Supplementary Figure 2. Regional association plots for the six loci identified in the meta-analysis.

The vertical axis indicates the $–\log_{10}$(P value) for the assessment of the association of each SNP with pancreatic cancer. Panels a to f show the plots for chromosome (chr) 1p13.2, 2p12, 3p12.3, 9q34.2, 13q12.2, or 13q22.1, in order. The colors indicate the LD (r^2) between each sentinel SNP and neighboring SNPs based on the JPT population in the 1000 Genomes Project Phase 3.

a. JPT subjects

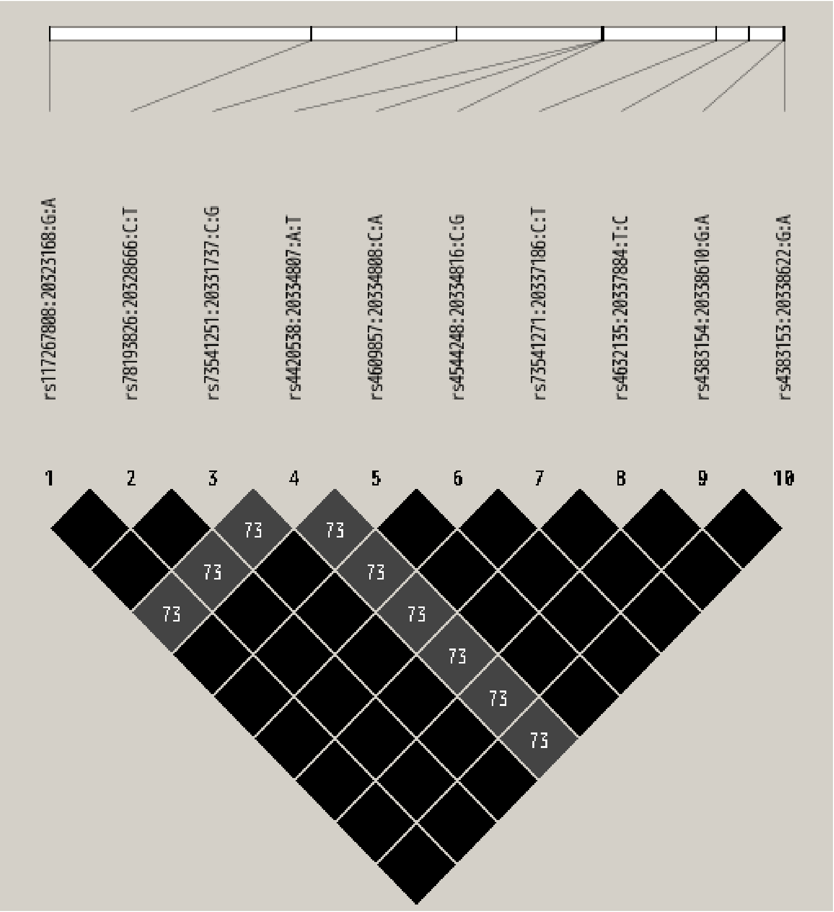

b. CEU subjects.

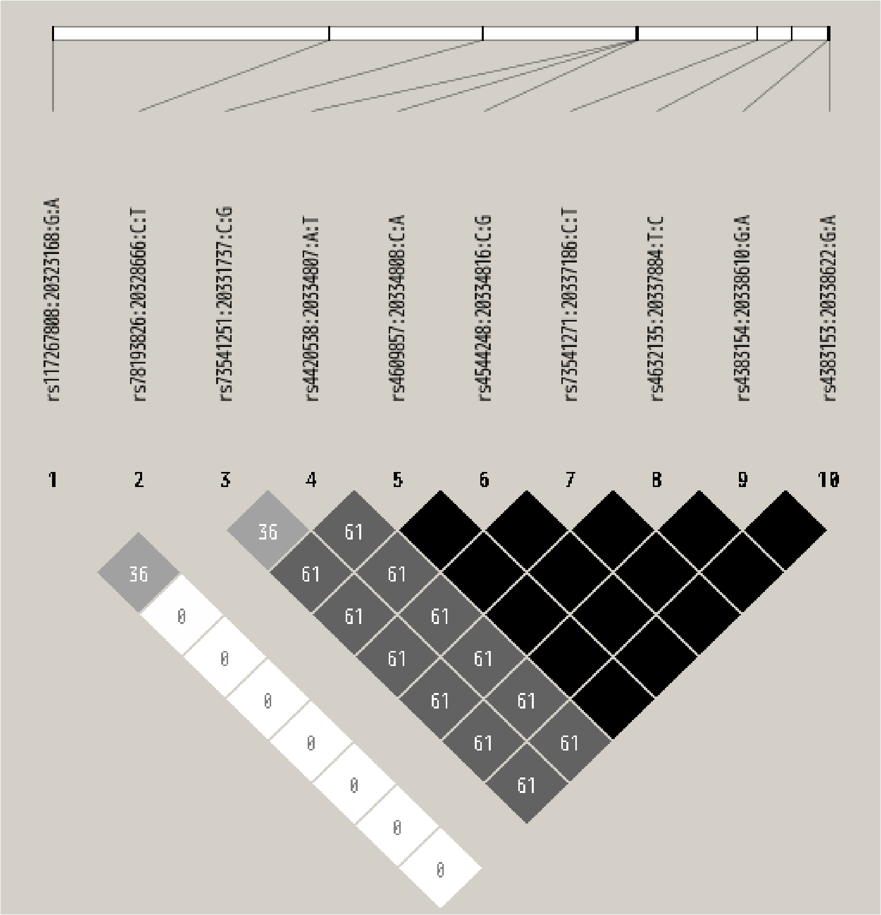

Supplementary Figure 3. LD maps of 10 SNPs with genome-wide significance at 16p12.3 based on 1000 Genomes.
Pairwise linkage disequilibrium r^2 values (white to black scales indicate low to high values), as determined with Haploview

a.

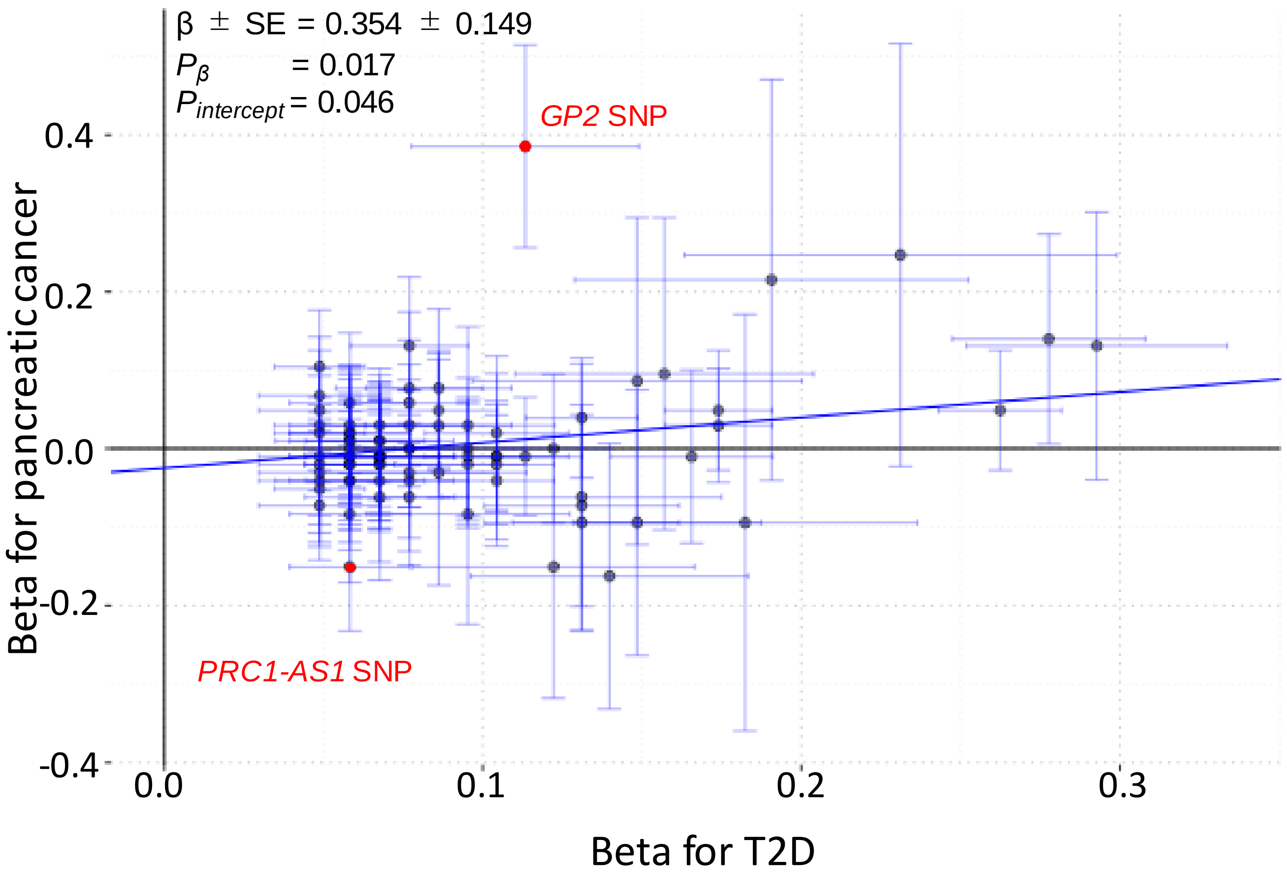
β ± SE = 0.354 ± 0.149
P_β = 0.017
$P_{intercept}$ = 0.046
GP2 SNP
PRC1-AS1 SNP
0.4
0.2
0.0
-0.2
-0.4
0.0
0.1
0.2
0.3
Beta for pancreatic cancer
Beta for T2D

b.

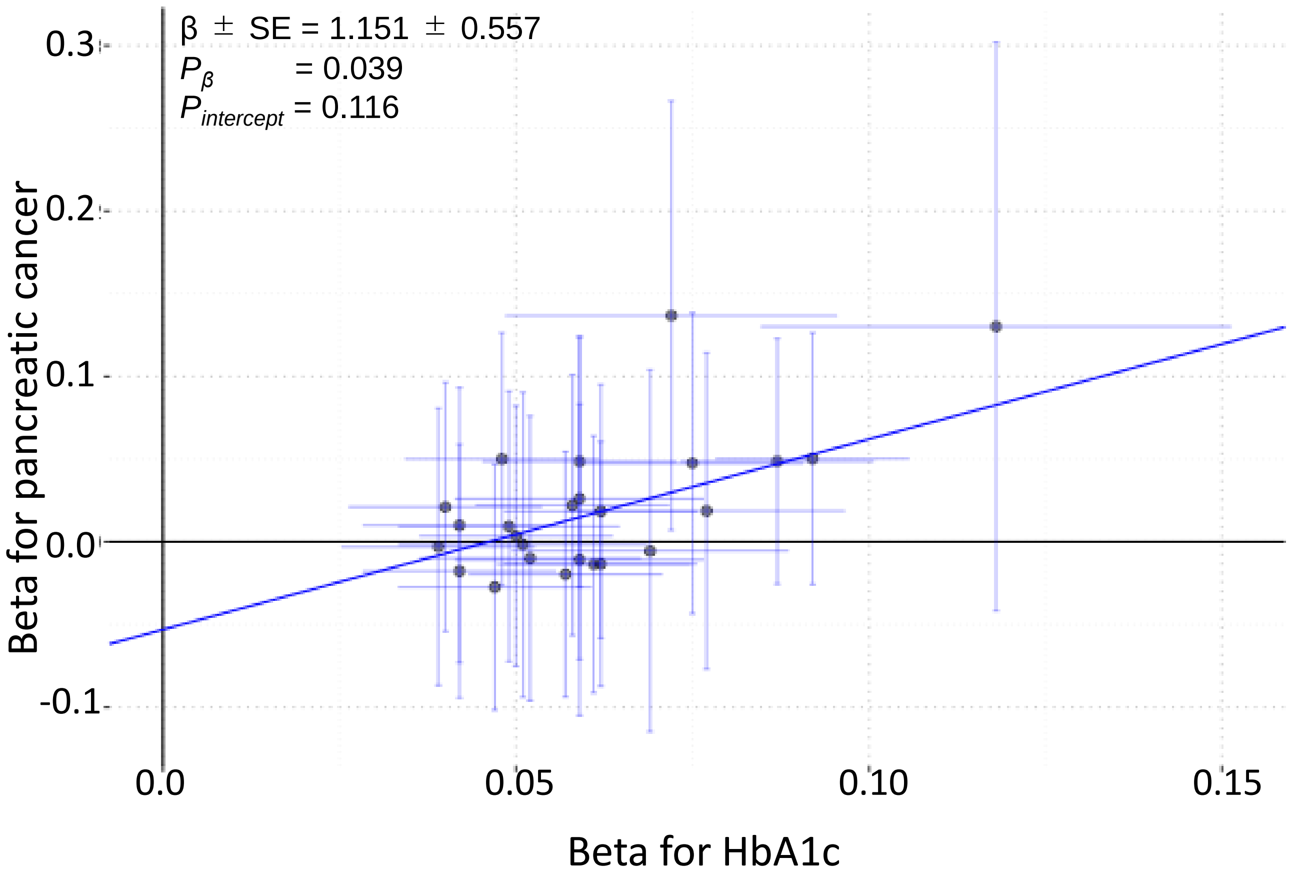

Supplementary Figure 4. Mendelian randomization (MR) analysis with the MR-Egger method for evaluating the relationship between T2D and pancreatic cancer in the Japanese population.

(a) The results for 82 T2D-associated SNPs. Beta values represent the log (OR) of T2D or pancreatic cancer per risk allele copy for each SNP. Red dots indicate the outlying SNPs detected by MR-PRESSO. (b) the results for 25 HbA1c-associated SNPs. Beta values represent the change in the rank-based inverse normal transformed values of the HbA1c level per risk allele copy for each SNP.

a.

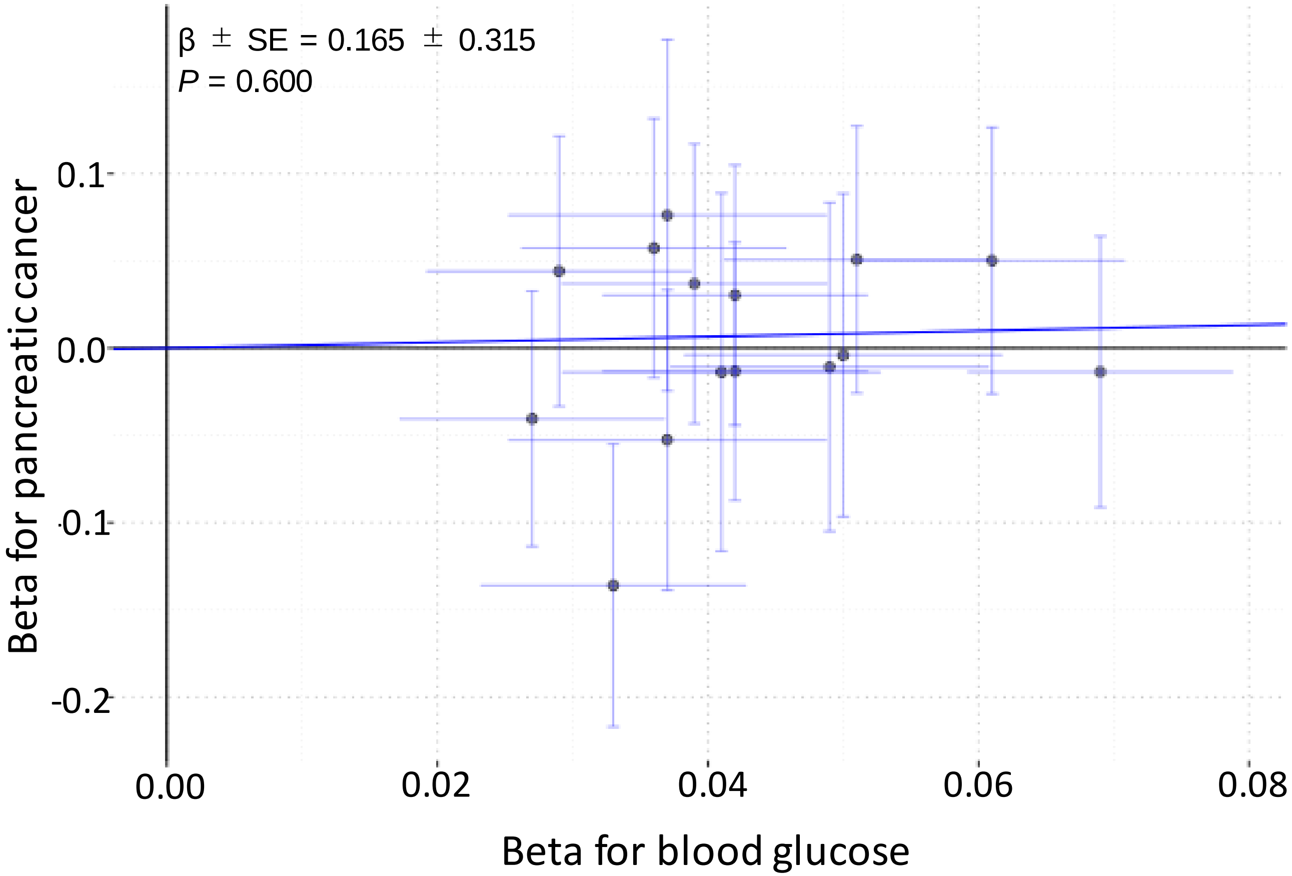
β ± SE = 0.165 ± 0.315
P = 0.600
0.1
0.0
-0.1
-0.2
0.00
0.02
0.04
0.06
0.08
Beta for pancreatic cancer
Beta for blood glucose

b.

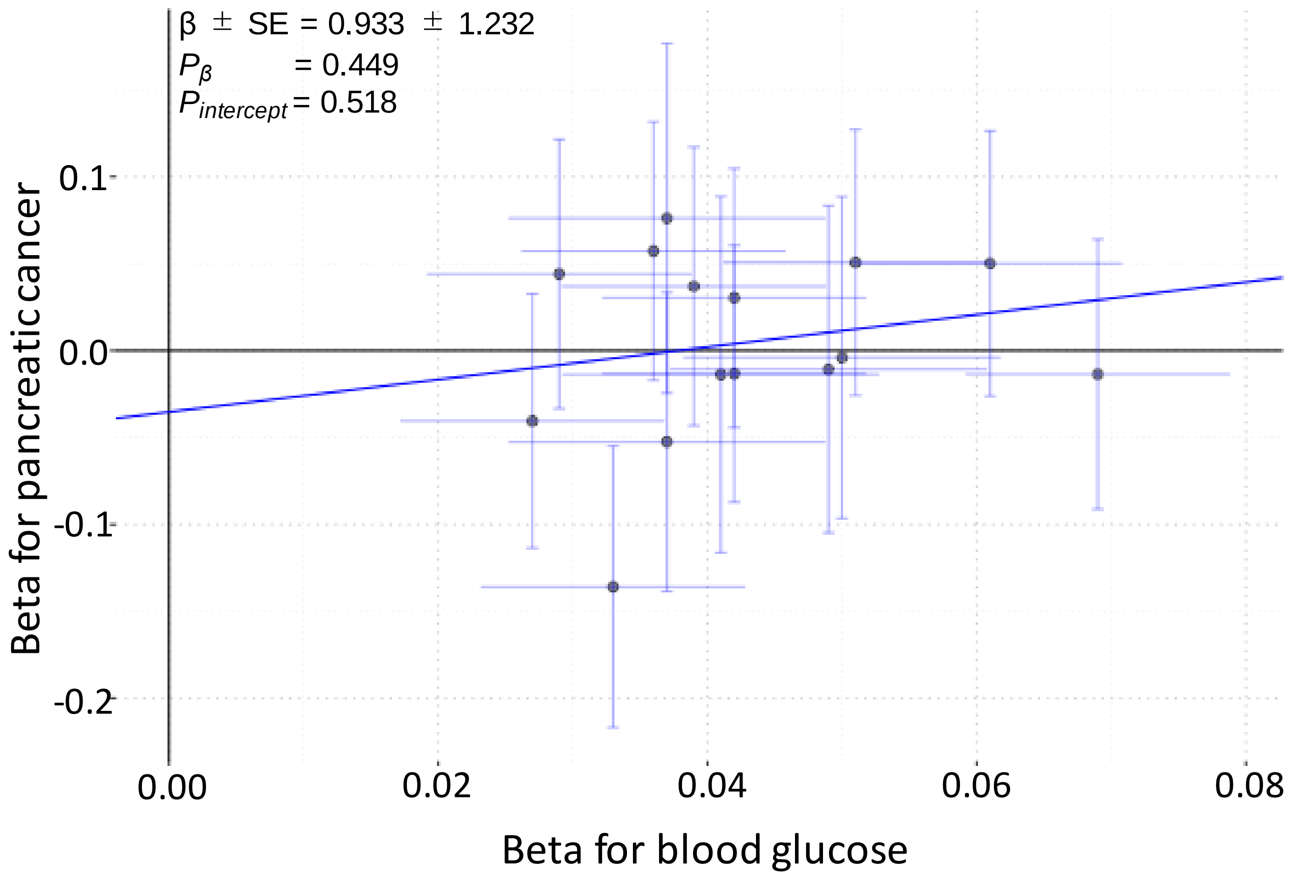
β ± SE = 0.933 ± 1.232
P_{β} = 0.449
$P_{intercept}$ = 0.518
Beta for pancreatic cancer
0.1
0.0
-0.1
-0.2
0.00
0.02
0.04
0.06
0.08
Beta for blood glucose

c.

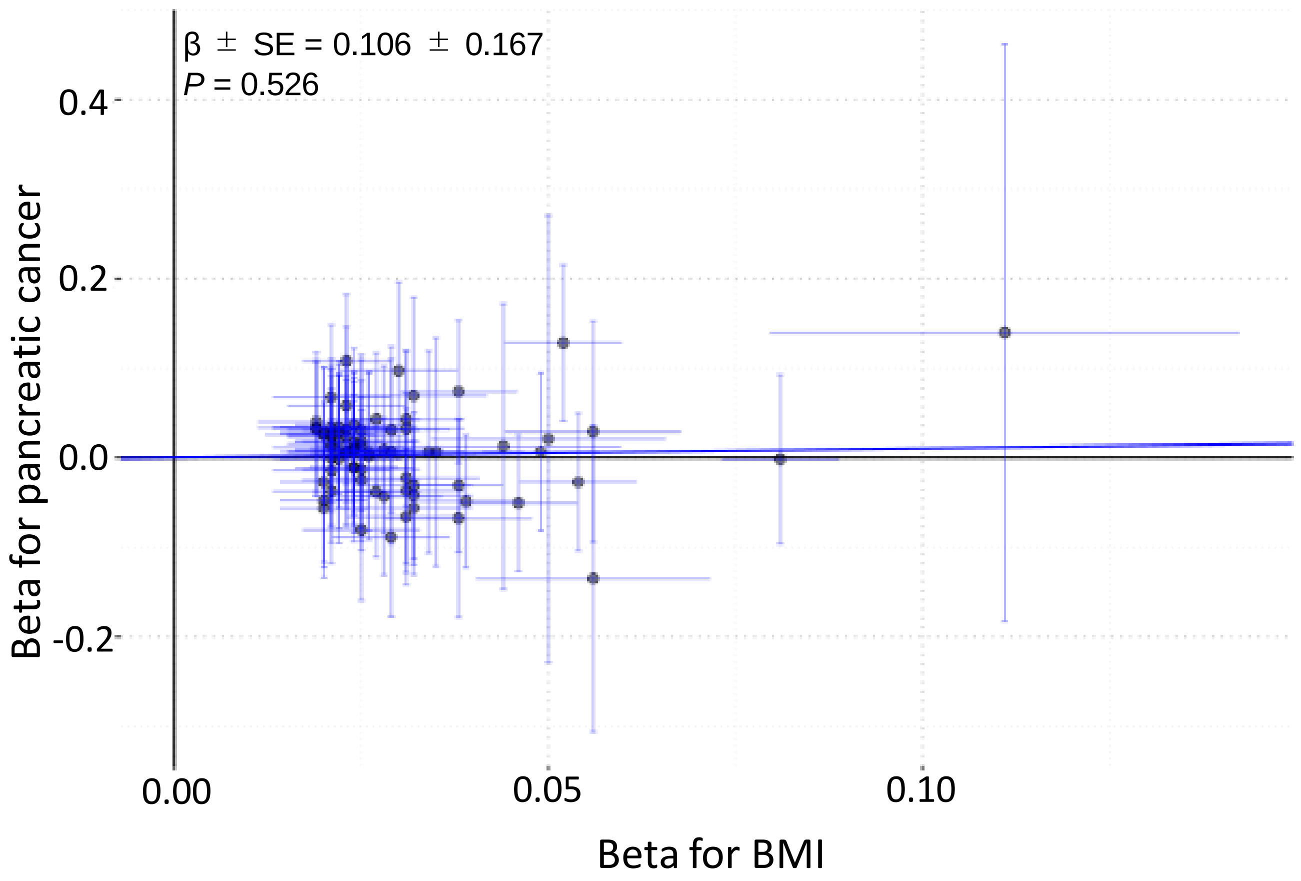
β ± SE = 0.106 ± 0.167
P = 0.526
0.4
0.2
0.0
-0.2
Beta for pancreatic cancer
0.00
0.05
0.10
Beta for BMI

d.

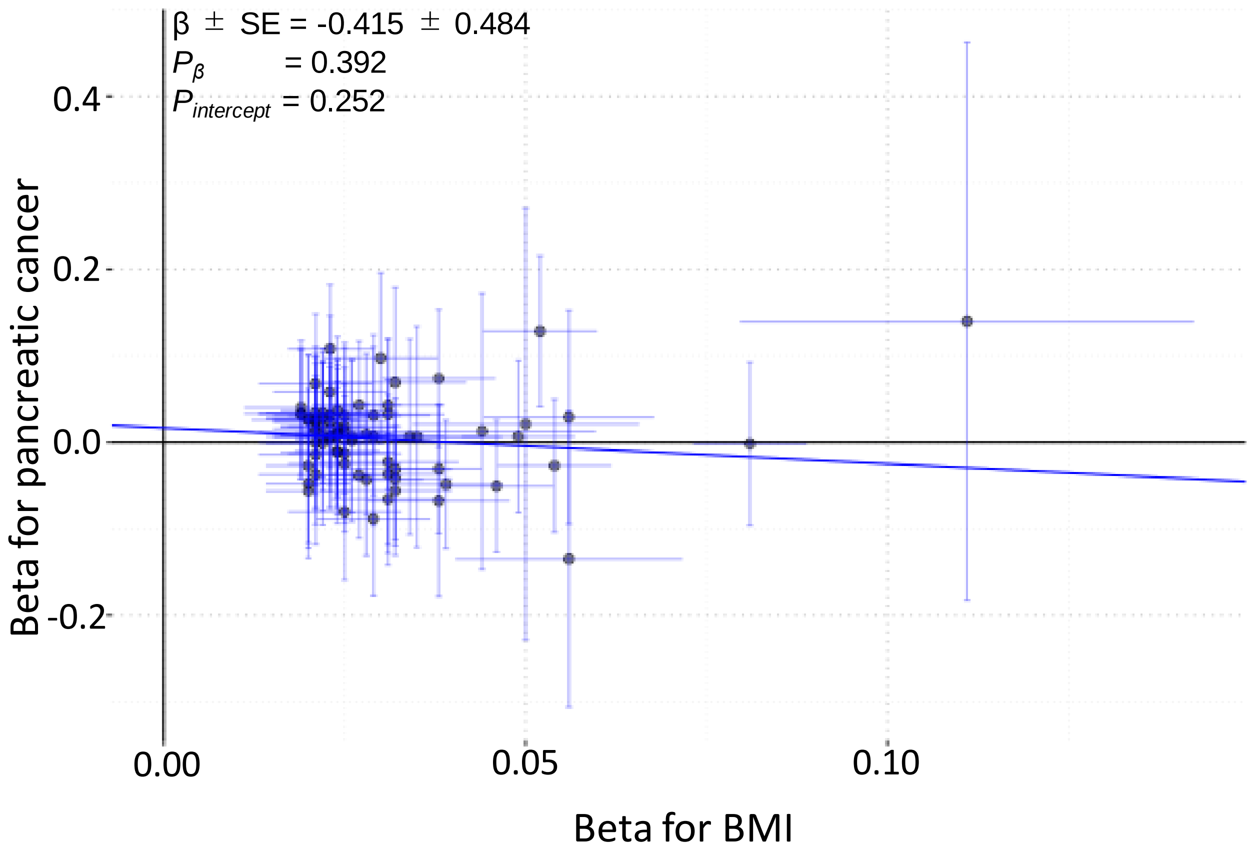

Supplementary Figure 5. MR analysis of the relationship between blood glucose, BMI, and pancreatic cancer in the Japanese population.

(a, b) The results with the IVW and Egger methods for 15 blood glucose-associated SNPs. Beta values represent the change in the rank-based inverse normal transformed values of the blood glucose level per risk allele copy for each SNP. (c, d) the results with the IVW and Egger methods for 76 BMI-associated SNPs. Beta values represent the change in the rank-based inverse normal transformed values of the BMI level per risk allele copy for each SNP.

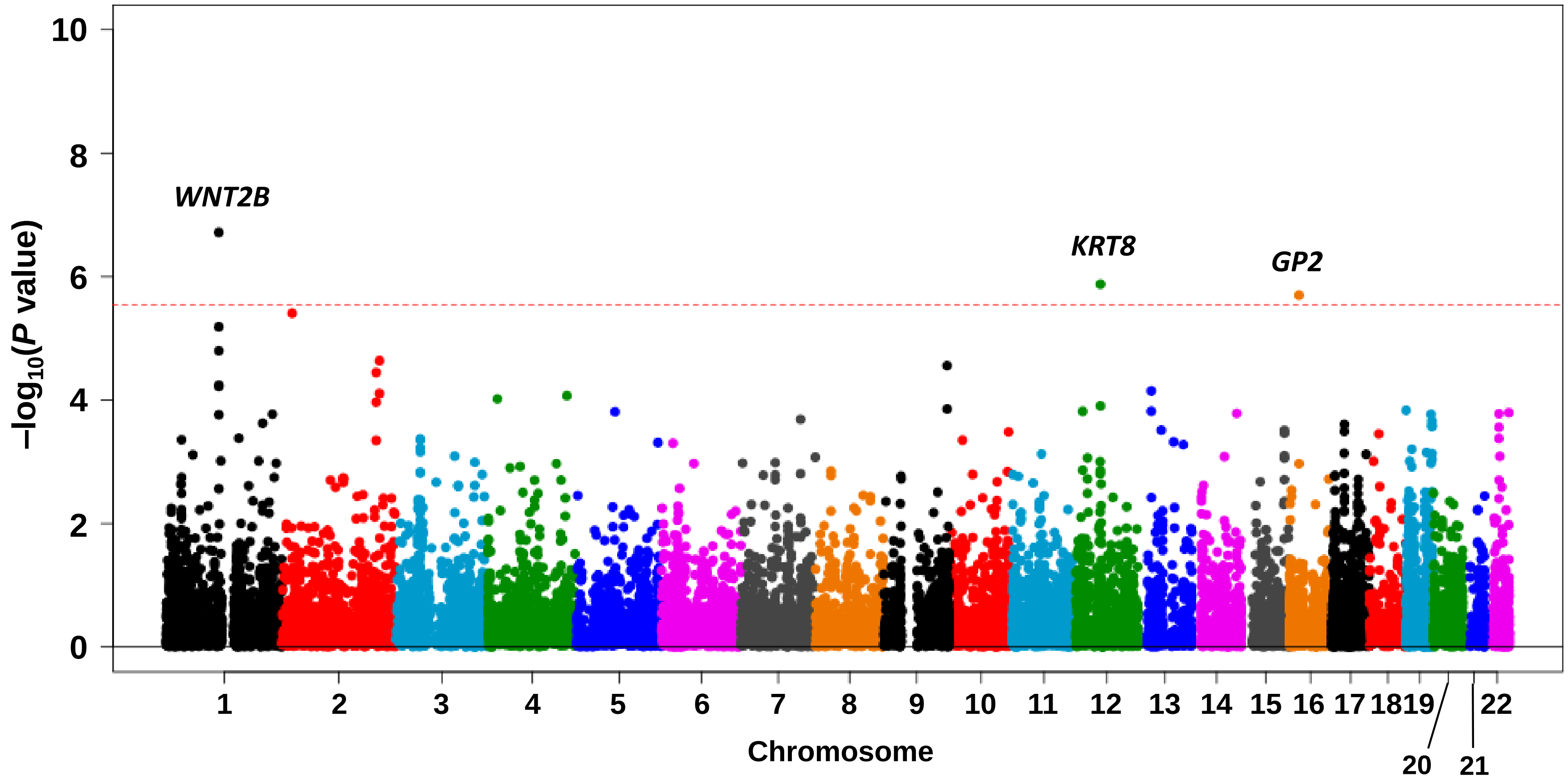

Supplementary Figure 6. Manhattan plot for the gene-based analysis.

The horizontal red line represents the genome-wide significance level ($\alpha = 2.84 \times 10^{-6}$).

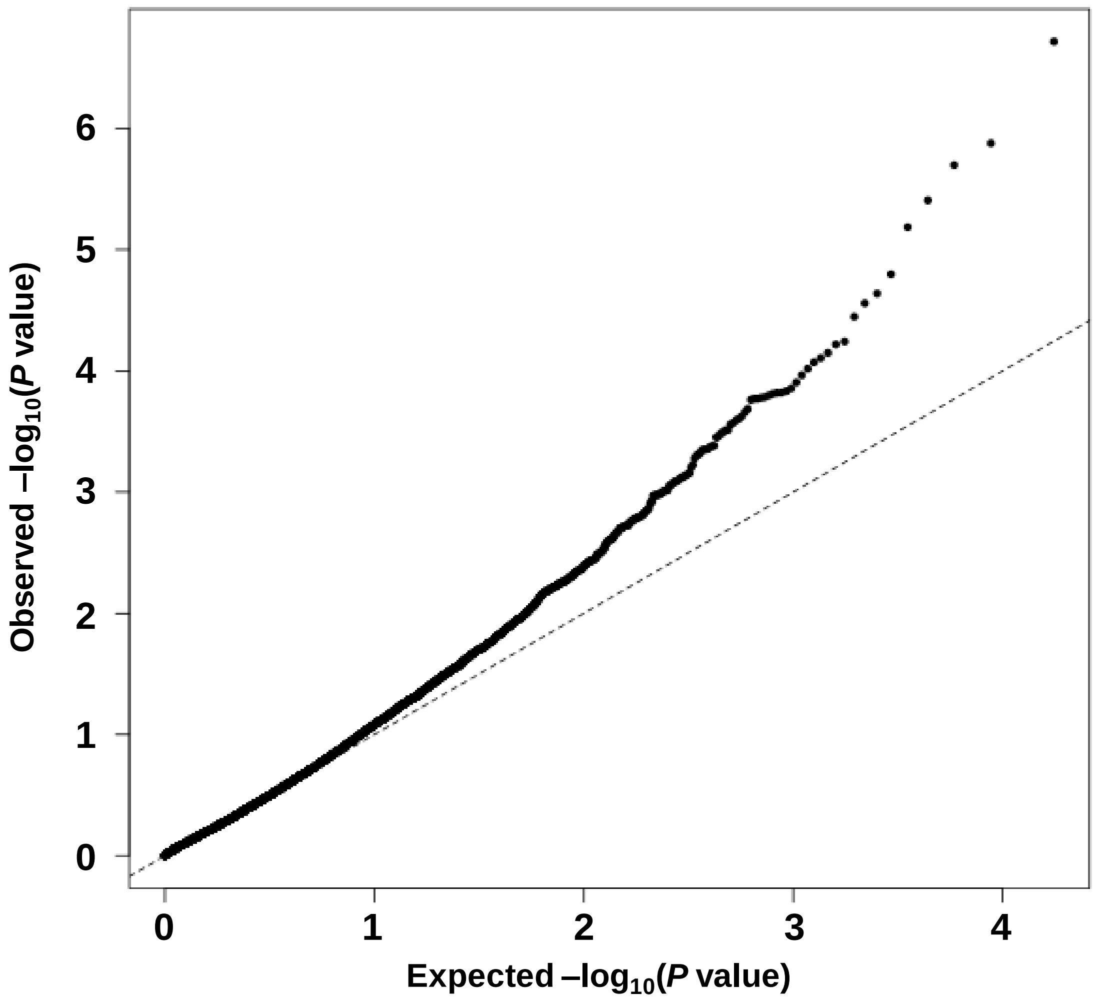

Supplementary Figure 7. Q-Q plot for the *P* values in the gene-based analysis.
The vertical and horizontal axes indicate the observed and expected $–\log_{10}$(*P* value) for the tests of association between genes and pancreatic cancer

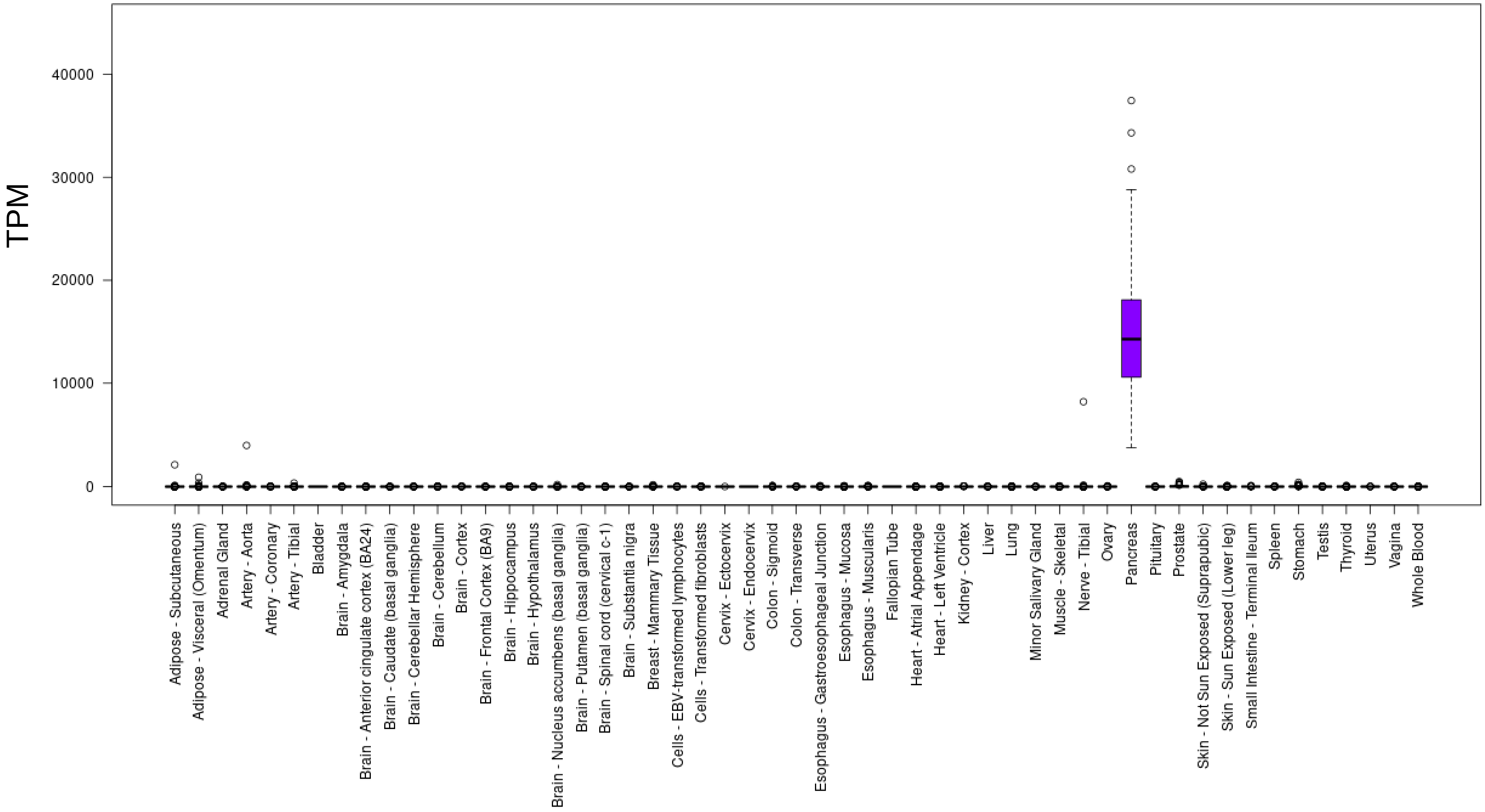

Supplementary Figure 8. GP2 (ENSG00000169347.12) differential gene expression illustrated across tissues.

The vertical axis indicates the TPM (transcripts per million) value, and the horizontal axis shows the tissues. The data used for the analyses described in this manuscript were obtained from the GTEx V7. Median, 25th, and 75th percentiles are shown in the box plot. The whiskers extend to the most extreme data point, which is no more than 1.5 times the interquartile range from the box.

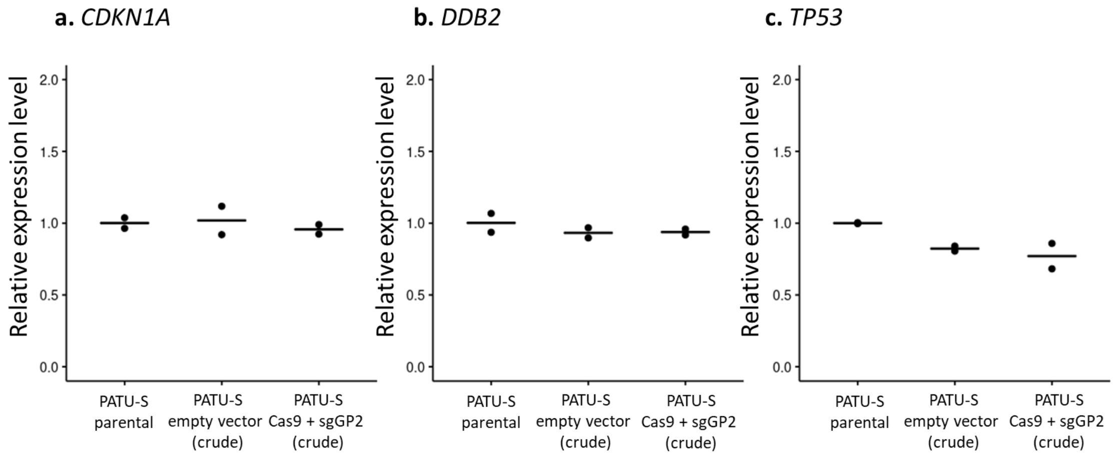

Supplementary Figure 9. Relative mRNA expression levels of genes in p53 pathway.

Expression levels of p53 pathway genes, such as *CDKN1A (a)* and *DDB2 (b)*, as well as *TP53 (c)* itself were determined by qRT-PCR under three different conditions: Patu-S parental cells, Patu-S cells transfected with the empty vector alone, and Patu-S cells transfected with the plasmid that we have used for genome editing in this study (N=2: technical replicates for each group). Representative results from three independent experiments are shown. Each dot indicates relative mRNA expression levels. The horizontal line represents mean levels of two data points.

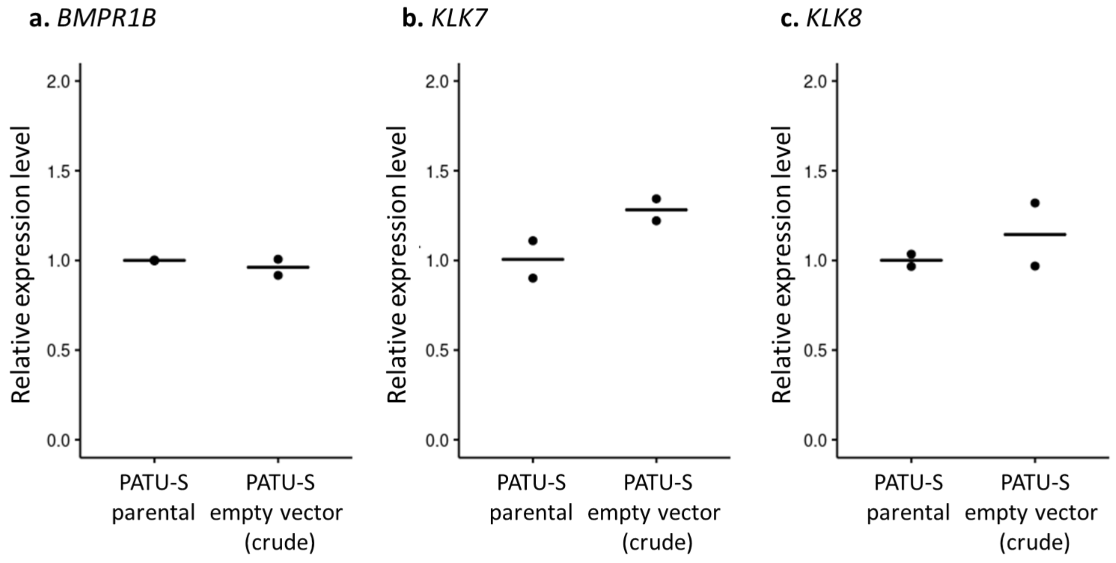

Supplementary Figure 10. Relative mRNA expression levels of genes in KRAS signaling pathway.

Expression levels of *BMPR1B* (a), *KLK7* (b), and *KLK8* (c) were determined by qRT-PCR under two different conditions: Patu-S parental cells and Patu-S cells transfected with the empty vector alone (N=2: technical replicates for each group). Representative results from three independent experiments are shown. Each dot indicates relative mRNA expression levels. The horizontal line represents mean levels of two data points.

Supplementary Note 1. Discussions on results of transient transfection experiments.

We did not look at changes of gene expression in clones established from WT GP2 cells transfected with the empty vector alone. Because the double strand breaks (DSBs) for genome editing occur through the transfection of the plasmid that we have used for genome editing in this study, we believe that off-target effects may not be induced by transfection of the empty vector alone, which cannot induce DSBs.

However, we performed additional transient transfection experiments to address the possible changes in gene expression of Patu-S cells transfected with the empty vector alone and off target effects. First, we examined the mRNA expression changes in two p53 pathway genes (*CDKN1A* and *DDB*2) as well as *TP53* itself under three different conditions: Patu-S parental cells, Patu-S cells transfected with empty vector alone, and Patu-S cells transfected with the plasmid that we have used for genome editing in this study. As shown in Supplementary Figure 9, no appreciable changes in the expression levels of those genes were noted among them. These findings suggest that possible off-target effects induced by the transfection of the empty vector as well as by DSBs do not affect the p53 pathway in our system, although we have used crude transfected cells in each group.

Additionally, we examined the mRNA expression changes in KRAS signaling pathway (*KLK7, BMPR1B*, and *KLK8*), under two different conditions: Patu-S parental cells and Patu-S cells transfected with the empty vector alone. No material changes in the expression levels of those genes were observed (Supplementary Figure 10). This experiment confirmed that possible off-target effects induced by the transfection of the empty vector alone do not affect KRAS signaling pathway, although we have used crude transfected cells in each group.

We have used the clones without mutation induced by transfection of the same genome editing plasmid as negative controls (GP2_WT). Using these clones as negative controls, we believe that we were able to see the GP2 mutation-specific gene expression changes in the clones of GP2_V425M, since CRISPR/Cas9-induced possible off-target effects (such as by unexpectedly induced mutations or by the p53 pathway activation[7]) may equally occur in the negative control clones and the GP2_V425M clones. To exclude the effects of clonal variation caused by selection bias, in addition, we have confirmed the expression changes of specific genes using 3 independent clones in each group in Figure 4E.Taken together, the data presented in the original paper (Figure 4E) exclude three possibilities: off-target effects through unexpectedly induced nonspecific mutations, non-specific p53 pathway activation

through DSBs alone, and transcriptional changes of nonspecific genes through GP2 mutation-independent clonal variations.

Supplementary Note 2. Additional details on the Biobank Japan Project and the population-based cohort studies.

The BioBank Japan Project

The Biobank Japan (BBJ, http://biobankjp.org) project was started in 2003 and collected DNA and clinical information from a total of 200,000 patients with at least one of 47 common diseases, including pancreatic cancer. These subjects were recruited from a collaborative network of 66 hospitals organized by 12 medical institutions in Japan (Osaka Medical Center for Cancer and Cardiovascular Diseases, the Cancer Institute Hospital of the Japanese Foundation for Cancer Research, Juntendo University, Tokyo Metropolitan Geriatric Hospital, Nippon Medical School, Nihon University School of Medicine, Iwate Medical University, Tokushukai Hospitals, Shiga University of Medical Science, Fukujuji Hospital, National Hospital Organization Osaka National Hospital, and Iizuka Hospital). The eligibility of the patients was determined by physicians at the hospitals. Overall, 422 patients with pancreatic cancer for whom genotype data were available were recruited from BBJ for this study. Clinical information was collected via a standardized questionnaire through a medical records survey. For the controls, we used quality control-accepted genotype data for 28,861 individuals from four population-based studies: the Japan Multi-Institutional Collaborative Cohort Study (J-MICC), the Japan Public Health Center-based Prospective Study (JPHC), the Tohoku Medical Megabank Project Organization (ToMMo), and the Iwate Tohoku Medical Megabank Organization (IMM). A separate manuscript with the results of this single-association analysis, along with analyses of 43 additional diseases, is under review (Ishigaki et al.)

Japan Multi-Institutional Collaborative Cohort Study (J-MICC study).

In the J-MICC study, 40,892 men and 51,750 women aged 35 to 69 years completed medical history questionnaires and donated blood samples at the time of the baseline survey, between 2004 and 2014[1]. The participants were recruited in 14 study areas throughout Japan among community dwellers, patients at the first visit to a cancer hospital, and health checkup examinees. For the present analyses, approximately 500 to 2,000 participants were selected from each study area, considering the number of respondents from each field and the geographical distribution of the subjects. All participants provided written informed consent. The ethics committees of Nagoya University (the affiliation of the principal investigator) and the other participating institutions approved the protocol for the J-MICC study. The following research institutions participated in the study: Chiba Cancer Center, University of Shizuoka,

Nagoya City University, Aichi Cancer Center, Nagoya University, Shiga University of Medical Science, Tsuruga Nursing University, Kyoto Prefectural University of Medicine, University of Tokushima, Kyushu University, Saga University, and Kagoshima University.

Japan Public Health Center-based Prospective Study (JPHC).

The JPHC samples were derived from a cohort of 33,736 residents in 9 public health center (PHC) areas who not only returned a self-administered questionnaire but also donated 10 mL of venous blood at the time of the baseline survey[2]. For the first sample selection step, we stratified the cohort by sex, 5-year age categories, and 9 PHC areas and then conducted random sampling, in which a similar proportion of subjects was selected from each stratum. Consequently, we determined 9,296 subjects for inclusion in the present GWAS. Before using the JPHC samples for genetic research, we obtained approval from the institutional review board of the National Cancer Center (Approval No.: 2011-044), Tokyo, Japan, and provided all eligible subjects the opportunity to refuse participation in the research.

The Tohoku Medical Megabank (TMM) Project (Tohoku Medical Megabank Organization (ToMMo) and Iwate Tohoku Medical Megabank Organization (IMM).

The TMM project is a reconstruction project from the Great East Japan Earthquake (2011) conducted by Tohoku University (http://www.megabank.tohoku.ac.jp/english/) and Iwate Medical University (http://iwate-megabank.org/en/)[3]. The TMM project encompasses two prospective cohort studies in Miyagi and Iwate Prefectures, Japan: the TMM Community-Based Cohort Study (TMM CommCohort Study) and the TMM Birth and Three-Generation Cohort Study (TMM BirThree Cohort Study). The TMM CommCohort Study is a population-based adult cohort study and recruited approximately 84,000 participants aged 20 years or older during 2013–2016. As of July 2017, the TMM BirThree Cohort Study had recruited approximately 74,000 participants, including fetuses and their parents, siblings, grandparents, and extended family members. All participants in the TMM project consented to genetic studies. Biospecimens (blood and urine) and medical data (questionnaires, blood and urine tests, and physiological measurements) were collected at the baseline examination. These samples and information are stored in the integrated biobank of the TMM project. DNA samples of the participants in the TMM CommCohort Study recruited in 2013 were analyzed by using the Illumina OmniExpressExome array (N=10,000). Information about age and sex was collected by using self-administered questionnaires and by

reviewing municipal basic resident registers. Of the 10,000 persons with available genotype data, 9,202 had their height and weight measured in a standard manner. For persons without the body height and weight measurements (N=798), values of these variables were obtained from self-reported questionnaires when available (N=703). The remaining 95 persons who had neither measured nor self-reported values were excluded from the analyses.

Supplementary Note 3. Additional details on studies included in the replication analysis

Japan Nationwide Collaborative Study Group for Early Pancreatic Adenocarcinoma (JEPA)

JEPA was a nationwide Japanese study group aimed at prospectively collecting clinical samples of early pancreatic adenocarcinoma patients, which started in 2015. The following institutions participated in the study: Cancer Institute Hospital, Chiba University, Higashiosaka City Medical Center, Hokkaido University, JA Onomichi General Hospital, Kagoshima University, Kanagawa Cancer Center, Kansai Rosai Hospital, Nagoya University, Osaka International Cancer Institute, Osaka Police Hospital, Osaka Rosai Hospital, Osaka University, Sakai City Medical Center, Tohoku University, Tokyo Women's Medical University, Toyonaka Municipal Hospital, University of Tokyo, Yamaguchi University, and Yokohama City University. This study was approved by the institutional review board of Osaka University (the affiliation of the principal investigator) and the other participating institutions approved the protocol for the study. Once the participant institution found patient(s) with early pancreatic adenocarcinoma [candidate(s) for this study], imaging diagnosis and histological/cytological diagnosis were performed, and the patient(s)' clinical samples were prospectively collected after obtaining written informed consent.

Nested case–control study within Japan Multi-Institutional Collaborative Cohort Study (J-MICC study)

In the J-MICC Study, 40,887 men and 51,744 women aged 35 to 69 years completed questionnaires on lifestyle and medical history and donated blood samples at the time of the baseline survey between 2004 and 2014[4]. The participants were recruited in 14 study areas throughout Japan among community dwellers, patients at the first visit to a cancer hospital, and health checkup examinees. The 14,569 controls for the BBJ GWAS were chosen considering the number of participants in each study area. For the replication analysis, we included 83 incident cases of pancreatic cancer reported through 2018. Three controls per case were selected, individually matching sex, age (same age), and study area to the corresponding case, and excluding the controls for the BBJ GWAS. All participants provided written informed consent. The ethics committees of Nagoya University (the affiliation of the principal investigator) and the other participating institutions approved the protocol for the J-MICC study. The following research institutions participated in the study: Chiba Cancer Center, University of Shizuoka, Nagoya City University, Aichi Cancer Center, Nagoya University, Shiga

University of Medical Science, Tsuruga Nursing University, Kyoto Prefectural University of Medicine, University of Tokushima, Kyushu University, Saga University, and Kagoshima University.

Case–cohort study within the Japan Public Health Center-based Prospective Study (JPHC)

The JPHC samples were derived from a cohort of 33,736 residents in nine public health center (PHC) areas who not only returned a self-administered questionnaire but also donated 10 mL of venous blood at the time of the baseline survey[2]. A case–cohort design was applied to perform a replication study in the JPHC study. After excluding those who were candidates to be used as controls in the BBJ GWAS and those with a past history of cancer, 23,228 subjects were potentially eligible for the replication study. After applying standard sample exclusion criteria, 2,510 randomly chosen subcohort subjects (including 17 incident cancer cases) and 68 incident pancreatic cases occurring outside of the subcohort were included in the analysis. Among these 2,493 non-cases and 85 cases, we conducted an ordinary prospective logistic regression analysis to estimate odds ratios and their standard errors. Before using the JPHC samples for genetic research, we obtained approval from the institutional review board of the National Cancer Center (Approval No.: 2011-044), Tokyo, Japan, and provided all eligible subjects the opportunity to refuse to participate in the research.

The Yale Shanghai Study

The Yale Shanghai Study is a population-based case–control study of pancreatic cancer in urban Shanghai, China[5]. The study was approved by the institutional human subjects review boards of the Shanghai Cancer Institute (Shanghai, China) and Yale University (New Haven, CT). All study participants were Shanghai residents aged 35 to 79 years. Blood samples were obtained from 761 cases (85%) and 794 controls (74%). Samples for the replication study included 740 patients with pathology-confirmed pancreatic cancer and 770 control subjects who were randomly selected from among Shanghai residents. Genotyping was performed at the National Cancer Institute using Illumina Global Screening Array.

Multiethnic Cohort Study

The Multiethnic Cohort Study (MEC) is a prospective study of over 215,000 men and women aged 45–75 years recruited from 1993 to 1996 from Los Angeles County and Hawaii, United States. The cohort design and baseline characteristics have been

described in detail previously[6]. The MEC includes primarily five racial/ethnic groups: European American, African American, Latino American, Japanese American, and Native Hawaiians. At enrollment, participants completed a baseline questionnaire assessing demographics, medical conditions, diet, lifestyle factors, and family history of cancer. Biological samples, primarily blood samples, were collected from approximately 70,000 cohort participants. Incident cancer cases in the cohort are identified via annual linkage to the National Cancer Institute's Surveillance, Epidemiology and End Results Program tumor registries in Hawaii and California.

For this replication study, we included Japanese Americans in a nested case–control study of pancreatic cancer in the MEC. Primary invasive pancreatic cancer cases were identified using ICD-O-3 code C25 and further restricted to those with pancreatic ductal adenocarcinoma (PDAC) histology. Controls were selected among MEC participants for whom DNA samples were available and without pancreatic cancer and matched 1:1 based on age, sex, and race/ethnicity. The case–control samples were genotyped using the 2M Multiethnic Genotyping Array (MEGA) from Illumina (San Diego, CA). In addition to the matched controls, we included additional controls with MEGA array data from previous GWAS in the MEC. Following quality control and exclusion of related individuals and those with missing covariate data, the analyzed dataset for this replication study included 183 pancreatic cancer cases and 3,597 controls. The Institutional Review Boards for the University of Southern California and the University of Hawaii approved this study.

Supplementary Reference:

1. Wakai, K. et al. Profile of participants and genotype distributions of 108 polymorphisms in a cross-sectional study of associations of genotypes with lifestyle and clinical factors: a project in the Japan Multi-Institutional Collaborative Cohort (J-MICC) Study. J. Epidemiol. **21**, 223-33 (2011).
2. Tsugane, S., Sawada, N. The JPHC study: design and some findings on the typical Japanese diet. Jpn. J. Clin. Oncol. **44**, 777-782 (2014).
3. Kuriyama, S. et al. The Tohoku Medical Megabank Project: Design and Mission. J. Epidemiol. **26**, 493-451 (2016).
4. Hamajima, N. et al. The Japan Multi-Institutional Collaborative Cohort Study (J-MICC Study) to detect gene-environment interactions for cancer. Asian. Pac. J. Cancer Prev. **8,** 317-23 (2007).
5. Risch, H.A. et al. Helicobacter pylori seropositivities and risk of pancreatic carcinoma. Cancer. Epidemiol. Biomarkers. Prev. **23**:172-178 (2014).
6. Kolonel L.N., et al. A multiethnic cohort in Hawaii and Los Angeles: baseline characteristics. Am. J. Epidemiol.**151**:346-357 (2000).
7. Schiroli, G. et al. Precise gene editing preserves hematopoietic stem cell function following transient p53-mediated DNA damage response. Cell. Stem. Cell. **24**, 551-565 (2019).
