# Supplementary material for: Genome-wide association meta-analysis identifies GP2 gene risk variants for pancreatic cancer
Source: Nat Commun. 2020 Jun 24;11:3175. doi: 10.1038/s41467-020-16711-w (PMC7314803; doi:10.1038/s41467-020-16711-w)
Supplement: Supplementary file 2 — Description of Additional Supplementary Files [file 41467_2020_16711_MOESM2_ESM.docx]

**Description of Additional Supplementary Files**

**File name:** Supplementary Data 1

**Description:** Cohort-specific information on genotyping, imputation and association testing

**File name:** Supplementary Data 2

**Description:** Allele frequency, effect size, and functional annotation for 10 SNPs with genome-wide significance at 16p12.3

**File name:** Supplementary Data 3

**Description:** Significantly differentially expressed genes (E-FDR < 0.10) in the GP2_V282M cells compared to the GP2_WT cells.

**File name:** Supplementary Data 4

**Description:** Significantly enriched hallmark gene sets identified in the GSEA.

**File name:** Supplementary Data 5

**Description:** Significantly enriched C6 oncogenic signatures identified in the GSEA

**File name:** Supplementary Data 6

**Description:** T2D-related SNPs most recently identified in the Japanese population by a GWAS (Suzuki et al., Nat Genet 2019) and the associations of these SNPs with pancreatic cancer risk in the present GWAS meta-analysis

**File name:** Supplementary Data 7

**Description:** Blood glucose-related SNPs most recently identified in the Japanese population by a GWAS (Kanai et al., Nat Genet, 2017) and the associations of these SNPs with pancreatic cancer risk in the present GWAS meta-analysis

**File name:** Supplementary Data 8

**Description** HbA1c-related SNPs most recently identified in the Japanese population by a GWAS (Kanai et al., Nat Genet, 2017) and the associations of these SNPs with pancreatic cancer risk in the present GWAS meta-analysis

**File name:** Supplementary Data 9

**Description:** BMI-related SNPs most recently identified in the Japanese population by a GWAS (Akiyama et al., Nat Genet, 2017) and the associations of these SNPs with pancreatic cancer risk in the present GWAS meta-analysis

**File name:** Supplementary Data 10

**Description:** Associations of pancreatic cancer with 19 SNPs with genome-wide significance that were reported in the previous PanScan and PanC4 consortia GWAS (Klein et al., Nat Commun, 2018).
